# Greater medial meniscus extrusion seen on ultrasonography indicates the risk of MRI-detected complete medial meniscus posterior root tear in a Japanese population with knee pain

**DOI:** 10.1038/s41598-022-08604-3

**Published:** 2022-03-19

**Authors:** Daisuke Chiba, Tomoyuki Sasaki, Yasuyuki Ishibashi

**Affiliations:** 1 Department of Orthopaedic Surgery, Hirosaki Memorial Hospital, Hirosaki, Japan; 2 Department of Orthopaedic Surgery, Hirosaki University Graduate School of Medicine, 5 Zaifu-cho, Hirosaki, Aomori 036-8562 Japan

**Keywords:** Cartilage, Magnetic resonance imaging, Ultrasonography

## Abstract

To elucidate the association between medial meniscus extrusion measured on ultrasonography (MME_US_) and the prevalence of medial meniscus posterior root tear detected on magnetic resonance imaging (MMPRT_MRI_). We recruited 127 patients (135 knees; 90 females; mean age: 64.4 ± 8.7 years old; mean BMI: 25.5 ± 3.4 kg/m^2^) in this cross-sectional study. All participants had medial knee pain without a knee trauma or surgery history. Knee osteoarthritis (KOA) severity was evaluated using Kellgren-Lawrence grade (KLG) scores. Patients with KLG scores 0–1 and ≥ 2 were classified in non-radiographic (non-ROA) and radiographic KOA (ROA) groups, respectively. MME_US_ was measured with patients in the supine position. Based on fat-suppressed T2-weighted images, MMPRT_MRI_ was defined as the presence of “Ghost meniscus sign” and “Cleft/truncation sign”, indicating an abnormal high signal intensity of a completely disrupted posterior root. MME_US_ was compared between MMPRT+ and MMPRT– patients using a non-paired t-test. Receiver operating characteristic (ROC) curves were used to determine the optimal cut-off MME_US_ to predict MMPRT+. The prevalence of MMPRT+ was 31.3% (25/80 knees) and 29.1% (16/55 knees) in the non-ROA and ROA groups. The MME_US_ of MMPRT+ patients were significantly greater than that of MMPRT– patients in both the non-ROA (5.9 ± 1.4 mm vs. 4.4 ± 1.0 mm, P < 0.001) and ROA (7.8 ± 1.3 mm vs. 6.3 ± 1.3 mm, P < 0.001) groups. ROC curves demonstrated that 5-mm and 7-mm MME_US_ were the optimal cut-off values in non-ROA (adjusted odds ratio: 6.280; area under the curve [AUC]: 0.809; P < 0.001) and ROA (adjusted odds ratio: 15.003; AUC: 0.797; P = 0.001) groups. In both early non-radiographic and established radiographic KOA stages, a greater MME_US_ was associated with a higher MMPRT_MRI_ prevalence.

## Introduction

The anterior and posterior meniscal roots are essential structures for anchoring the menisci to the tibial plateau and maintaining the meniscal function of articular cartilage protection. These bony attachments of the menisci allow the circumferential collagen fibers in the meniscal body to disperse axial loads into hoop stresses that redistribute the forces applied to the knee joint during daily activities^1,2^. Specifically, the posterior root of the medial meniscus is rigidly attached to the tibia, thereby rendering the meniscal body less mobile compared to the other roots. Therefore, the posterior root of the medial meniscus is more vulnerable to not only traumatic damage such as hyperflexion or squatting but also degenerative changes, resulting from a higher incidence of root tears^3,4^. Medial meniscus posterior root tear (MMPRT), defined as an avulsion injury or radial tear occurring within 10 mm from a bony attachment, has gained the attention of clinicians and surgeons to deal with knee symptom management^5–7^. MMPRT substantially disrupts the ability of medial meniscus to anchor the stretching of circumferential collagen fibers in a radial direction; the medial meniscus radially extrudes toward the outside of the knee joint during the allocation of axial loading force^3^. Consequently, MMPRT not only alters loading stress distribution to increase peak contact pressure and decrease contact area in the medial compartment of the tibiofemoral joint^3,5,8^, but also influences joint arthrokinematics by increasing the level of lateral tibial translation and medial compartment excursion^9^. Intra-articular derangement derived from MMPRT eventually leads to a rapid progression of osteoarthritis (OA) or spontaneous osteonecrosis in the affected knee joint^5,10–12^. More recently, the concept of early knee OA has drawn the interest of clinicians and researchers in the early diagnosis of knee OA to prevent the progression of OA changes^13,14^. Accordingly, the early diagnosis of MMPRT prior to definitive knee OA development significantly contributes to the improvement of patient quality of life in terms of locomotive function. Due to the lack of highly sensitive or specific history and physical findings^3^, the use of magnetic resonance imaging (MRI) has been valuable in MMPRT detection based on the presence of a linear defect around the posterior bony insertion of meniscal roots on axial or coronal view^15–17^. Moreover, other important findings are observed on sagittal view, such as the “Ghost meniscus sign”, which indicates the complete absence of an identifiable triangular meniscal body as a high signal replacing the normal dark meniscal signal; a normal meniscus is seen on the immediately adjacent images^3,5,15,17–19^. Nevertheless, MRI has limitations such as increased cost and an increased duration of examination. Therefore, the use of a more convenient alternative examination modality to validate the risk of MMPRT would be helpful in the outpatient room, for instance. Ultrasonography (US) has several advantages over MRI; US provides physicians with an inexpensive, non-invasive, quick, and real-time assessment of the knee joint^20–22^. Several studies have reported that US demonstrates a relatively high sensitivity and specificity in meniscal pathology detection^20,23,24^. On the other hand, the presence of artifacts which originate from adjacent bone surface makes the examiners have difficulty to detect the inner meniscal margins^25^. Therefore, the available US data on the direct detection of posterior root rupture in the medial joint space are scarce. Limited previous studies have reported that US could indirectly detect greater medial meniscus extrusion (MME) in a knee joint with MMPRT in the biomechanical^26^ or clinical setting^27^. More recent cohort study reported that a knee joint with a greater MME measured by US (MME_US_) had a higher risk of knee OA development or aggravation regardless of MMPRT occurrence^22^. Thereafter, MME_US_ has the potential to indirectly reveal the presence of MMPRT in patients having knee symptoms with a predisposition for knee OA development or aggravation. Unfortunately, it is difficult to validate the sensitivity of MME_US_ to indicate the presence of MMPRT due to a lack of previous data. Thus, we aimed to cross-sectionally evaluate how the value of MME_US_ can indicate the presence of MMPRT on MRI (MMPRT_MRI_), dividing the patients who have medial knee joint pain into non-radiographic knee OA and radiographic knee OA. We hypothesized that the greater value of non-weight bearing MME_US_ with patients in the supine position would be associated with a higher prevalence of MMPRT_MRI_.

## Methods

### Study participants

The current study was approved by the ethical committee of Hirosaki Memorial Hospital (IRB No. 2020-13). Informed consent was obtained from all participants. All methods were performed in accordance with the relevant guidelines and regulations (Declaration of Helsinki). Study participants visited our hospital from December 2019 to December 2020 presenting with medial knee joint pain during daily activities such as walking, climbing and descending stairs, and sitting and standing. During this period, 186 patients visited our outpatient clinic. We excluded the patients without MRI or US data, and those with a history of other rheumatic disease such as rheumatoid arthritis and that of previous knee trauma (i.e., ligament injury or fracture) or surgery. Finally, we recruited 127 patients (135 knees; 90 females; mean age: 64.4 ± 8.7 years old; mean BMI: 25.5 ± 3.4 kg/m^2^) for the current study.

Weight bearing anteroposterior knee radiographs were acquired with film-focus distance of 100 cm, 68 kV, and 20 mAs. Plain radiographs of the affected knee were taken in an orthostatic position with the knee semi-flexed, corresponding to Rosenberg view. According to the Kellgren-Lawrence grade (KLG)^28^, single orthopaedic surgeon (DC, 12-year experience) assigned participants to the non-radiographic knee OA (Non-ROA: KLG 0–1) or radiographic knee OA (ROA: KLG 2–4) group. The intra-rater reliability (k) for determining KLG was 0.821 for the right knee and 0.805 for the left knee^29^.

### Ultrasonographic measurement of MME

**Figure 1:**
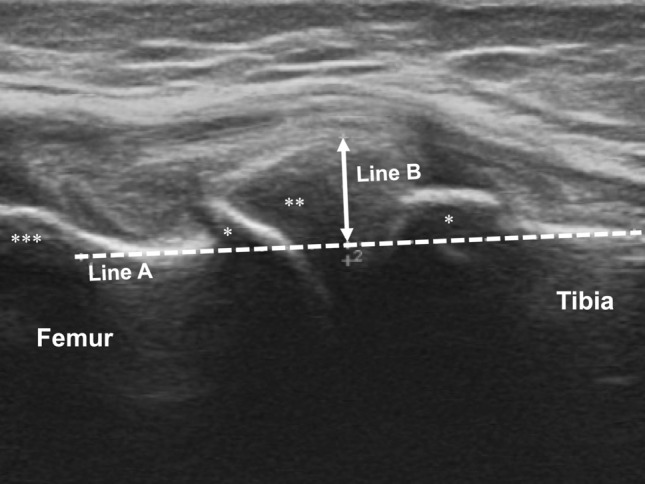
Ultrasonographic evaluation of medial meniscus extrusion. *Osteophyte, **Medial meniscus, ***Medial femoral epicondyle. Line A was drawn to connect the cortex of both the femur and tibia, thereby tracing the femoral cortex at the bottom of the medial femoral epicondyle. Line B was drawn perpendicularly from the bottom of Line A to the most medially extruded part of the medial meniscus. Line A was drawn through the osteophyte bases to avoid the bony interference of osteophytes throughout the length of Line B. Finally, the length of Line B (mm) was measured as the medial meniscus extrusion_._

All participants underwent measurement of non-weight bearing MME_US_ in the supine position. The ultrasound probe (12 MHz, ARIETTA Prologue, Hitachi Aloka Medical, Tokyo, Japan) was placed at the center of the medial knee joint space, with the knee joint fully extended and in neutral rotation. MME_US_ was measured on ultrasonographic image where the medial meniscus was displayed with a hypoechoic band of the medial collateral ligament^30^ and the downslope of the medial femoral epicondyle (Fig. 1). First, a line was drawn connecting the cortex of both the femur and tibia (Line A); thereafter, a line perpendicular to Line A was drawn from the bottom of Line A to the most medially extruded part of the medial meniscus (Line B, Fig. 1). Finally, the length of Line B was measured as the MME_US._ Regarding the technique used to draw Line A, we ignored the existence of osteophytes and drew Line A through the osteophyte bases to avoid the bony interference of osteophytes for Line B. Additionally, on the femoral side of Line A, this line should trace the femoral cortex at the bottom of the medial femoral epicondyle (Fig. 1). In the outpatient room, single orthopaedic surgeon (DC) acquired US image and measured MME_US_ before the patients underwent the plain radiographs of affected knee. Regarding the intra-rater reliability of DC, intraclass correlation coefficient [ICC] (1.1) was 0.977 (95% CI 0.942–0.991)^22^. Based on total of 18 knee-OA patients, ICC (1.1) was measured by reading the US images twice to wait one-week interval between first and second measurement of MME.

### MRI evaluation of medial meniscus tear

**Figure 2:**
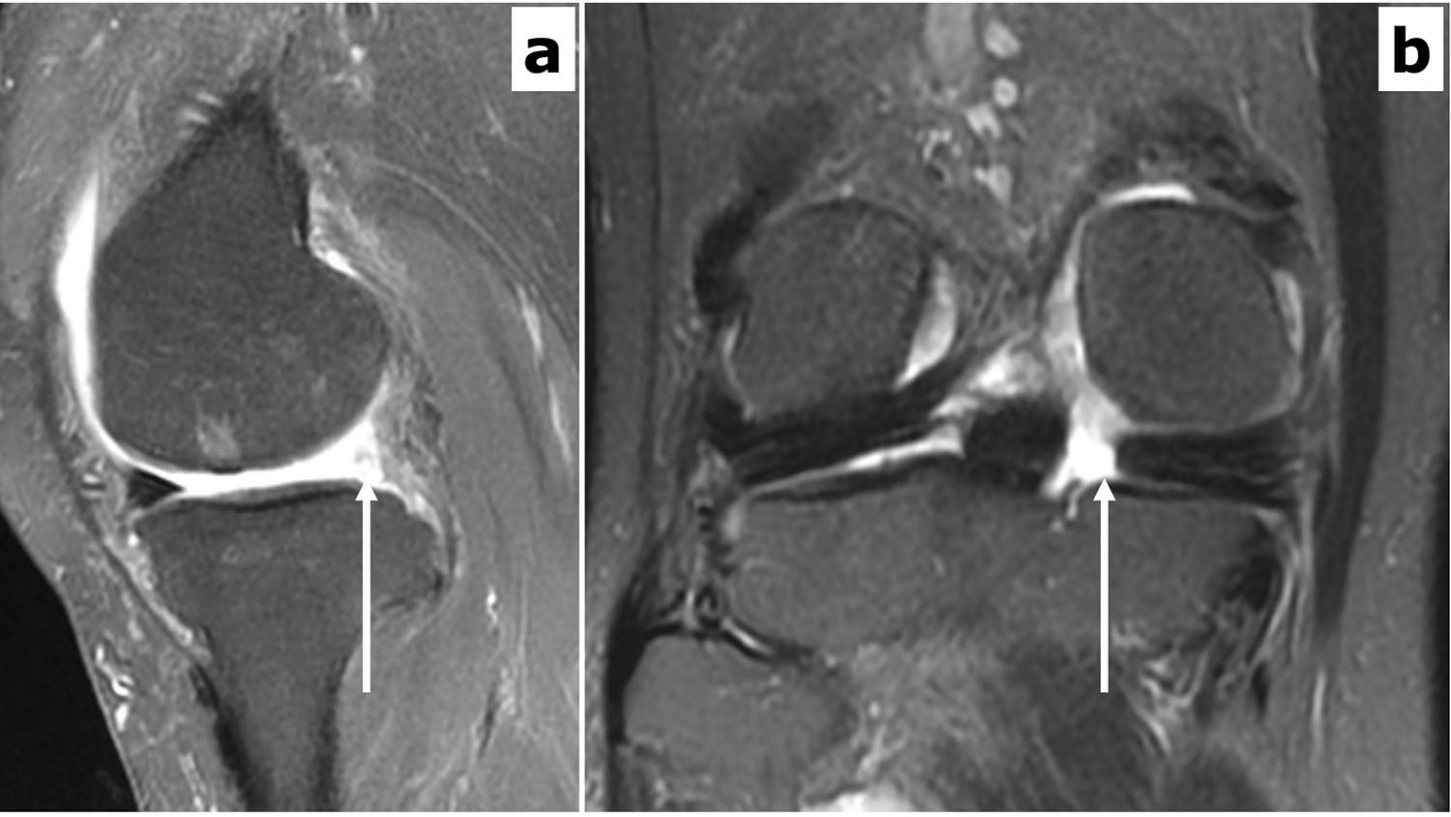
Ghost meniscus sign on a fat-suppressed T2-weighted sagittal image. (**a**) Sagittal view showing ghost meniscus sign, (**b**) coronal view showing a defect of the medial meniscus posterior root. An abnormally high signal, indicating the absence of an identifiable meniscal body, is detected in place of the normal dark meniscal signal at the posterior horn of the medial meniscus (arrow).

All participants underwent 1.5-T MRI for the affected knee joint (Optima MR360, GE Healthcare). The knee joint was kept immobilized by customized frame during examination with 10° knee flexion and externally rotated at 10–15°. A T2-weighted image with fat-suppressed sequence was used to evaluate MMPRT^31–35^ with the following imaging sequence: coronal T2 with fat suppression (TR/TE[ms]: 2200/90, field of view: 200 × 200 mm, Matrix 320 × 224, Layer thickness: 4.0 mm, Gap: 1.0 mm) and sagittal T2 with fat suppression (TR/TE[ms]: 2000/32, field of view: 200 × 200 mm, Matrix 320 × 256, Layer thickness: 4.0 mm, Gap: 1.0 mm) Based on the sagittal images, the presence of MMPRT was defined using the ghost meniscus sign, which indicates the complete absence of an identifiable triangular meniscal body as a high signal replacing the normal dark meniscal signal seen on the immediately adjacent images (Fig. 2a)^5,17,19^. Furthermore, on coronal MRI, cleft/truncation sign (vertical linear defect) was defined as the other reference of MMPRT (Fig. 2b). If the current subjects had both ghost meniscus and cleft/truncation signs, they were defined as having MMPRT. Regarding the diagnostic performance of MMPRT, both ghost meniscus (96.7–100%) and cleft/truncation sign (90–100%) have been reported to demonstrate a high sensitivity^16,17^. Radiologist in our hospital finally diagnosed the presence of MMPRT_MRI_, blinded to the clinical information of current subjects. Regarding the other forms of medial meniscus tear, further classification was performed as longitudinal, radial, horizontal, oblique, or complex tear^36^. Moreover, the severity of meniscal tears was determined by WORMS Score: 0 = intact; 1 = minor radial tear or parrot-beak tear; 2 = non-displaced tear; 3 = displaced tear; 4 = complete maceration/destruction^37^.

### Statistical analysis

All statistical analyses were conducted using SPSS version 24 (SPSS Inc., Chicago, IL, USA). Continuous variables of demographic data and MME_US_ between patients with and without the MMPRT_MRI_ were compared by non-paired t-test. A receiver operating characteristic (ROC) curve was drawn to determine the optimal cut-off to predict the prevalence of the MMPRT_MRI_ in each of non-ROA or ROA group, respectively. Using the optimal cut-off of MME_US_, a logistic regression analysis was conducted with the prevalence of the MMPRT_MRI_ as the dependent variable, and with the optimal cut-off of MME_US_ as the independent variable, adjusted for age, sex, and body mass index. A P-value < 0.05 was considered statistically significant.

## Results

The prevalence of positive MMPRT (MMPRT+) was 31.3% (25/80 knees) and 29.1% (16/55 knees) in the non-ROA and ROA groups, respectively. The prevalence of MMPRT+ was not significantly different between the groups. In the non-ROA group, the mean age of patients in the MMPRT+ group was significantly higher than that of patients in the negative MMPRT (MMPRT−) group (Table 1). MME_US_ in MMPRT+ patients were significantly greater than that in MMPRT– patients in both the non-ROA (5.9 ± 1.4 mm vs. 4.4 ± 1.0 mm, P < 0.001) and the ROA groups (7.8 ± 1.3 mm vs. 6.3 ± 1.3 mm, P < 0.001, Table 1). Regarding the other form of medial meniscus tear, 116 knees (91.3%) had degenerative complex tear pattern including a component with horizontal pattern often communicating with the inferior meniscus surface on at least two image slices^38^. Twenty-four (20.7%) out of 116 knees having degenerative meniscus tear showed severe meniscal maceration. Only one case had definitive flap tear which was arthroscopically resected. All MMPRT+ patients had the comorbidity of degenerative medial meniscus tear around its central to posterior portion. Ten knees (7.9%) showed no medial meniscus tear.

**Table 1:** Current participant demographic characteristics.

		MMPRT− (N = 55)			MMPRT+ (N = 25)			*P *value
Non-ROA	Age, year	61.3	±	9.3	67.6	±	7.4*	0.004
	Sex (F/M)	29	–	26	18	–	7	0.105
	BMI, kg/m^2^	24.6	±	2.9	25.3	±	3.3	0.332
	MME, mm	4.4	±	1.0	5.9	±	1.4*	< 0.001

		MMPRT− (N = 39)			MMPRT+ (N = 16)			P-value
ROA	Age, year	66.1	±	8.0	66.1	±	7.0	0.992
	Sex (F/M)	31	–	8	12	–	4	0.714
	BMI, kg/m^2^	26.5	±	3.8	26.0	±	3.1	0.644
	MME, mm	6.3	±	1.3	7.8	±	1.3*	< 0.001

*P ≤ 0.05, non-paired t-test; MME, medial meniscus extrusion; ROA, radiographic knee osteoarthritis (Kellgren-Lawrence grade ≥ 2).

**Figure 3:**
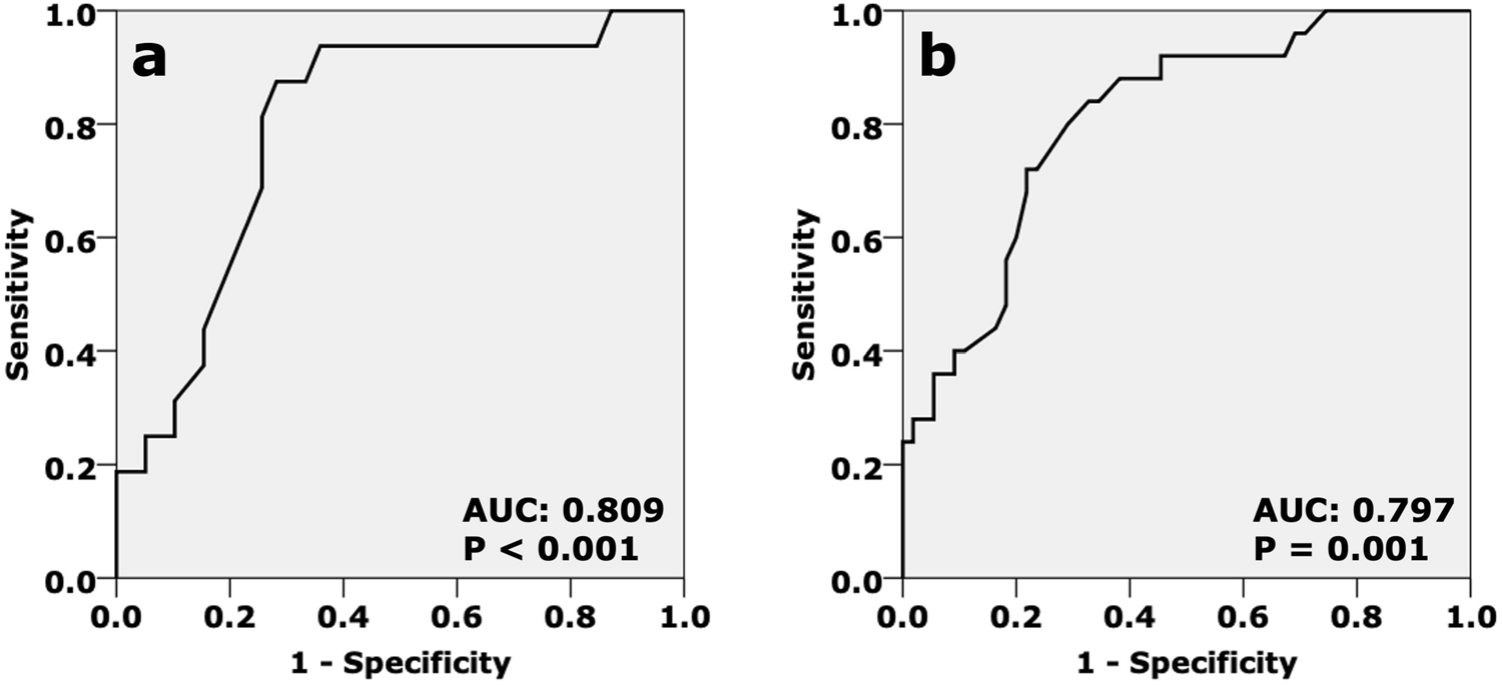
Receiver operating characteristic curve to predict the ghost meniscus sign on a fat-suppressed T2-weighted sagittal image. (**a**) Non-ROA group; (**b**) ROA group; AUC: area under curve; ROA: radiographic knee osteoarthritis (Kellgren-Lawrence grade ≥ 2).

Overall, the ROC curve demonstrated that the MME_US_ value could significantly predict the prevalence of MMPRT+ (AUC: 0.736; 95%CI: 0.647 to 0.824; P < 0.001). In addition, the ROC curve consistently demonstrated that the MME_US_ value could significantly predict the prevalence of MMPRT+ in both the non-ROA (AUC: 0.809; 95%CI: 0.710 to 0.907; P < 0.001) and the ROA (AUC: 0.797; 95%CI: 0.669 to 0.925; P = 0.001) group, respectively. Based on the ROC curve, the optimal cut-offs of MME_US_ in the non-ROA and ROA groups were 5 mm (Sensitivity: 76.0%; 95%CI: 66.6% to 85.4% and Specificity: 73.6%; 95%CI: 63.9% to 83.3%) and 7 mm (Sensitivity: 81.3%; 95%CI: 71.0% to 91.6% and Specificity: 74.4%; 95%CI: 62.9% to 85.9%), respectively (Fig. 3).

Multiple logistic regression analysis showed that a 5-mm MME_US_ was significantly associated with the prevalence of MMPRT+ in the non-ROA group (adjusted odds ratio: 6.280; 95%CI: 1.854 to 21.277; P = 0.003). Similarly, a 7-mm MME_US_ was associated with the prevalence of MMPRT+ in the ROA group (adjusted odds ratio: 15.003; 95%CI: 3.923 to 69.205; P = 0.001, Table 2).

**Table 2:** Logistic regression analysis to elucidate the relationship between the cut-off value of medial meniscus extrusion and the prevalence of the ghost meniscus sign.

Cut-off value		Model	B	*P *value	OR	95% CI
MME 5 mm	Non-ROA	Crude	2.277	< 0.001	9.750	3.119–30.477
		Adjusted	1.837	0.003	6.280	1.854–21.277
MME 6 mm	Non-ROA	Crude	1.897	0.002	6.667	1.971–22.553
		Adjusted	1.542	0.024	4.674	1.222–17.877
	ROA	Crude	2.345	0.030	10.435	1.249–87.144
		Adjusted	2.678	0.018	14.554	1.588–133.344
MME 7 mm	ROA	Crude	2.531	0.001	12.567	2.958–53.390
		Adjusted	2.708	0.001	15.003	3.253–69.205

B: regression coefficient; MME: medial meniscus extrusion; OR: odds ratio; ROA: radiographic knee osteoarthritis (Kellgren-Lawrence grade ≥ 2), 95%CI: 95% confidence interval. The MME cut-off values of 5 mm in ROA and 7 mm in non-ROA group are not significantly associated with the prevalence of ghost meniscus sign.

## Discussion

The most important finding of the current study is that a greater MME_US_ was associated with a higher prevalence of MMPRT_MRI_ based on a positive finding of the ghost meniscus and cleft/truncation signs, which corresponds to the complete rupture of the posterior root of the medial meniscus on sagittal and coronal fat-suppressed T2-weighted MRI. Notably, the current finding was consistently significant in patients with medial knee pain both with and without definitive radiographic OA changes defined by the KLG. Moreover, the cut-off value of MME_US_ differed according to the stage of radiographic knee OA.

The prevalence of MMPRT in the current study was 31.3% (25/80 knees) and 29.1% (16/55 knees) in the non-ROA and ROA groups. This prevalence of MMPRT was higher than the previous epidemiological data, ranged from 10.1 to 27.8%^39–41^. Interestingly, Bin et al.^39^ and Hwang et al.^41^ from Korea reported relatively similar prevalence of MMPRT with the current study. Asian people are more likely to experience MMPRT due to their lifestyle of frequent squatting and sitting on the floor with the legs deeply folded^5^. A greater MME_US_ was consistently associated with a higher prevalence of MMPRT_MRI_, thereby verifying our study hypothesis. In accordance with the significant loss of meniscal function resulting from MMPRT, some previous cohort studies reported that patients with medial meniscus posterior root injury on MRI^42,43^ demonstrate a greater MME on the corresponding MRI. In line with these previous cohort study findings, our study findings showed that the use of US is compatible with that of MRI in terms of measuring MME for MMPRT prevalence determination. Furthermore, US is likely to be clinically relevant for the easily validation of the risk of MMPRT in patients with medial knee joint pain in the outpatient clinic.

However, there is insufficient evidence to connect the relationship between MME_US_ and the prevalence of MMPRT at this point. A cadaveric biomechanical study reported that the resection of the posterior root of the lateral meniscus affected the degree of MME_US_^26^. For knees with a total resection of the posterior root, a greater MME_US_ was observed in comparison to that of knees with a partial resection. Specifically, when an axial load was applied, MME_US_ was significantly greater than when a non-axial load was applied; the latter was simulated as non-weight bearing condition^26^. Karpinski et al. conducted a study with a similar design to that of the current study to elucidate the relationship between the prevalence of MMPRT_MRI_ and the values of MME measured by both US and MRI^27^. They observed a greater MME value in participants who had MMPRT in the knee OA population with a relatively early stage of KLG 0–2. This study^27^ also evaluated the alterations between the weight and non-weight bearing conditions of MME_US_. Interestingly, the results of Karpinski et al. conflicted with those of Rowland et al.^26^ wherein weight bearing condition did not change the value of MME_US_^27^. Based on these limited US data, there remains a controversy with regard to the relationship between the value of MME_US_ and the prevalence of MMPRT. The biomechanical effect of MMPRT on MME_US_ would change in accordance with the severity of cartilage degeneration^44,45^, lateral or medial meniscus involvement, and weight or non-weight bearing condition. Moreover, to improve inter-rater reliability, future studies should be conducted to determine a consistent method of MME_US_ measurements.

The most important limitation of previous US studies^26,27^ is the small sample size; the reliability of MME_US_ was not high enough to determine the prevalence of MMRPT. Thus, the lack of evidence makes it difficult for musculoskeletal healthcare providers to consider the values of MME_US_ that validate the prevalence of MMPRT at various stages of knee OA. Based on our study data, with a relatively large sample size, we can progress in the further discussion of applying the optimal cut-off of MME_US_ to determine the prevalence of MMPRT. In the current non-radiographic OA population with KLG 0–1, a 5-mm MME_US_ is the optimal cut-off to detect MMPRT on fat-suppressed T2-weighted sagittal and coronal MRI. In contrast, a 7-mm MME_US_ cut-off is optimal for detecting MMPRT in the definitive radiographic OA population with KLG ≥ 2. Compared to the findings of Karpinski et al., the current cut-off values are greater; notably, the upper error bar of the supine position MME_US_ was between 5 and 6 mm in the study by Karpinski et al.^27^ In other words, further discussion would be needed to conclude which MME_US_ value is best to detect the prevalence of MMPRT, and future large-sample studies will shed light on the detailed mechanism of the effect of meniscus root rupture on the deterioration of meniscal function.

Our study had several limitations. First, we could not validate whether the current study participants had grossly visible MMPRT using arthroscopy. Second, the US evaluation was performed only in the supine position with a non-weight bearing condition. Therefore, we did not discuss how the weight bearing condition affects MME_US_ based on the current data. Third, the sample size of the advanced knee OA stage (KLG 3–4, N = 20 knees) was relatively small. Finally, the design of the current study was cross-sectional, and therefore we could not establish a cause-effect relationship between MME_US_ value and MMPRT development or aggravation. Despite these limitations, the current study emphasizes the clinical relevance of MME_US_ in detecting the prevalence of MMPRT by healthcare providers. The use of US can potentially aid in the evaluation of posterior root meniscus rupture in an outpatient room.

## Conclusion

The current cross-sectional study elucidated the association between MME_US_ and the prevalence of MMPRT. For the patients having medial knee joint pain, a greater MME_US_ was associated with a higher prevalence of MMPRT on fat-suppressed T2-weighted MRI. MME_US_ ≥ 5 mm can be a risk factor for MMPRT in the patients with non-radiographic knee OA and MME_US_ ≥ 7 mm can be a risk factor for MMPRT in the patients with definitive radiographic knee OA.
